# Quadriceps muscle force magnitude and control in the knee scheduled for arthroplasty versus the contralateral knee: A cross-sectional study in patients with end-stage osteoarthritis

**DOI:** 10.1186/s43019-026-00305-9

**Published:** 2026-01-27

**Authors:** Thiago Lemos, Yan R. Razuck, Gustavo H. Halmenschlager, Conrado T. Laett, Sidney C. Silva, Alan P. Mozella, José C. S. Albarello

**Affiliations:** 1 Laboratório de Pesquisa Neuromuscular e Fisiologia do Exercício, Divisão de Ensino e Pesquisa–DIENP, Instituto Nacional de Traumatologia e Ortopedia Jamil Haddad–INTO, Avenida Brasil 500, Caju, Rio de Janeiro, RJ CEP 20940-070 Brasil; 2 Graduate Program in Rehabilitation Sciences, Centro Universitário Augusto Motta – UNISUAM, Rio de Janeiro, Brasil; 3 Center for Knee Surgery, Instituto Nacional de Traumatologia e Ortopedia – INTO, Rio de Janeiro, Brasil

**Keywords:** Orthopedics, Neuromuscular signs and symptoms, sensory-motor performance, Rehabilitation, Biomechanics

## Abstract

**Background:**

Knee osteoarthritis (KOA) is a leading cause of musculoskeletal disability. Beyond the well-established impairment of reduced strength, deficits in force control (steadiness and complexity) may also influence functional performance. This cross-sectional study investigated quadriceps strength, force steadiness and complexity in patients with KOA and their associations with functional performance.

**Methods:**

A total of 48 patients scheduled for unilateral knee arthroplasty performed maximal voluntary isometric contraction in both limbs. A 2-s window from the trial containing the peak torque was used to compute quadriceps strength (average torque, AT), force steadiness (coefficient of variation, CV), and force complexity (sample entropy, SE; detrended fluctuation analysis alpha exponent). Functional performance was assessed via sitting-to-standing, single-leg stance, Timed Up and Go, and 30-s sit-to-stand tests. Comparisons between involved and contralateral limbs used analysis of variance (ANOVA) models, accounting for prior surgery in contralateral knees. Linear regression analyzed associations between functional performance and the lateral symmetry index (LSI) of AT and SE.

**Results:**

Results showed significant differences between limbs for AT (*P* < 0.001, *η*^2^ = 0.074) and SE (*P* = 0.041, *η*^2^ = 0.046), with the involved knee exhibiting lower strength and higher complexity. Regression revealed a positive association between sitting-to-standing and 30-s sit-to-stand performance and LSI–AT (*βs* are equal to −0.337 and 0.336, respectively; *P* < 0.027), but no other links were found.

**Conclusions:**

KOA is associated with between-limb asymmetries in quadriceps strength and force complexity, with the involved knee exhibiting deleterious alterations. Nonetheless, force complexity was not correlated with functional performance.

## Background

Knee osteoarthritis (KOA) is one of the most prevalent musculoskeletal disabilities [1]. Characterized by inflammation, pain, and joint stiffness, KOA impairs the ability to perform daily activities, significantly reducing patients’ quality of life [2–4].

Patients with KOA consistently show marked structural and functional muscle impairments. These include severe weakness of the quadriceps and hamstrings [5], arthrogenic muscle inhibition that prevents full muscle activation [6], and poor muscle quality and altered biochemical composition [7, 8]—all of which are major determinants of physical function in advanced disease. Along with these factors, other muscle function characteristics, such as force control (steadiness and complexity), could also influence the progression of KOA.

Force steadiness is defined as the ability to maintain stable submaximal force [9] and is typically quantified as the variability of force during sustained contractions. Lower-limb force steadiness is impaired in various conditions [10–12] and is linked to functional decline in older adults, as well as in orthopedic and neurological patients [13, 14]. In patients with KOA, force steadiness is impaired relative to control patients [15–17]. This impairment, assessed in the more symptomatic knee during submaximal isometric and isokinetic contractions, was normalized following arthroplasty [17] but showed no association with functional gait measures [18].

Force complexity, however, quantifies the temporal/dynamic interactions across neuromuscular structures and regulatory feedback mechanisms [19], typically assessed via temporal regularity (e.g., sample entropy [20]) and long-term statistical dependency measures (e.g., detrended fluctuation analysis alpha exponent [21]). Evidence of reduced quadriceps force complexity (i.e., reduced force entropy and/or increases in alpha exponent) has been found with aging and frailty [22, 23] and after fatiguing contractions [21]. Moreover, increases in quadriceps force complexity were observed during maximal contractions in the anterior cruciate ligament (ACL)-injured knee, compared with the contralateral knee [20, 24]. Clinically, diminished force complexity reflects a loss of system resilience that impedes adaptability [25, 26], leading to reduced autonomy, poor rehabilitation outcomes, and greater vulnerability to age-related decline.

Despite the relevance of measuring force complexity for understanding muscle function, there is a scarcity of investigations using these measures in patients with KOA [27]. In addition, prior research on KOA and force steadiness suffers from methodological inconsistencies [15, 17, 18].

This study investigates the effects of end-stage KOA on between-limb asymmetries in quadriceps force magnitude and control and their relationship with functional performance. The end-stage osteoarthritic knee is typically defined by advanced radiographic disease (Kellgren–Lawrence grade ≥ 3 or Ahlbäck grade > III) combined with a high symptom burden [28, 29] that indicated a need for total knee arthroplasty. The contralateral knee, in contrast, is the less symptomatic knee with lower-grade impairment. Understanding the impact of end-stage KOA on these neuromuscular dimensions could provide relevant information for prognosis and rehabilitation.

## Methods

### Participants and ethical concerns

This cross-sectional study was conducted between September 2023 and March 2025 and approved by the institutional ethics committee (approval no. 64171022.3.0000.5273; 7 December 2022). All participants provided written informed consent prior to study participation.

Patients with end-stage KOA who were scheduled for unilateral primary total knee arthroplasty (TKA) were invited to participate in the study. They were referred to the hospital research unit during pre-admission, on the day before surgery. Eligible participants were adults over > 18 years of age, of both sexes, referred for unilateral TKA owing to radiographically confirmed KOA (Ahlbäck grade ≥ 3 [30]) and clinical indications, specifically persistent severe pain and significant functional limitation. Exclusion criteria included: (1) cardiovascular, metabolic, or musculoskeletal conditions that could hinder participation or (2) a history of orthopedic surgery on the involved knee (i.e., the knee scheduled for TKA).

A convenience sample of 56 participants was initially assessed. Four were excluded owing to poor signal quality or technical difficulties during data acquisition, and another four were excluded because of prior surgical intervention on the involved knee. Thus, data from 48 patients (31 females and 17 males) were included in the final analysis. Among these, 18 had a history of contralateral knee surgery (17 TKAs and one tibial screw placement), while the remaining 30 had no prior knee surgery.

### Outcomes

*Patient history*: The demographic, anthropometric, and clinical data were recorded or obtained through medical records.

To perform the functional tests described below, all participants are required to perform the tests either barefoot or wearing flat shoes to ensure consistency. If a participant requires an assistive device to complete a task or if physical assistance is needed, the test is considered failed, and the worst score is given.

*Sitting-to-standing test*: Adapted from Franchignoni et al. [31]. Test performance was scored on a three-point scale (2, normal; 1, moderate impairment; and 0, severe impairment/unable to perform), as follows: a score of 2 was given if the individual stood without using their hands and stabilized independently; a score of 1 was given if the participant succeeded but required hand support; and a score of 0 was given if the individual was unable to stand without assistance or required multiple attempts despite hand use.

*Single-leg stance test*: Also adapted from Franchignoni et al. [31], the test was performed with the involved knee. Two attempts were recorded, with the best trial used for analysis. A score of 2 (normal) was given if the participant maintained balance for 20 s, a score of 1 (moderate impairment) was given if the duration was less than 20 s, and a score of 0 (severe impairment/unable to perform) was given if the task could not be performed [32].

*The Timed Up and Go (TUG)*: The test assesses mobility by measuring the time required for a participant to stand up from a chair, walk 3 m, turn around, walk back, and sit down at their usual pace [33].

*The 30-s sit-to-stand test*: This test records the maximum number of repetitions completed within 30 s [34]. If the participant cannot complete the full duration, both the number of repetitions and elapsed time are recorded. The normalized metric of repetitions per second was used for analysis—for example, for a patient who performed 15 repetitions in 30 s, the normalized score was 0.5.

**Fig. 1:**
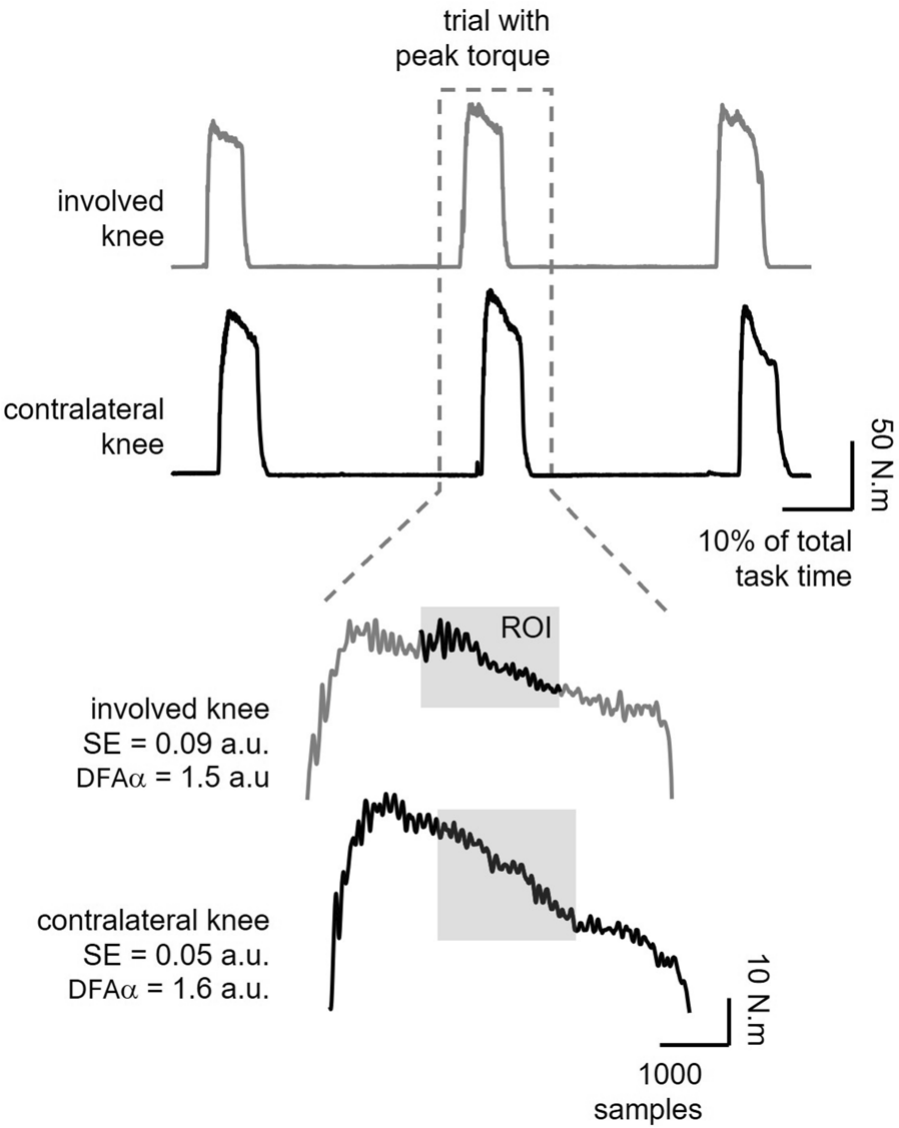
Torque signals during maximal isometric voluntary contraction from the involved (gray line) and contralateral knees (black line) in a representative participant (male, 61 years old). Upper panel: among the three contractions performed, the trials with peak torque (highlighted by the dotted-line square) were selected. Lower panel: from the selected trial, a 2-s region of interest (ROI, the central portion of the contraction, excluding its initial and final phases; see gray shading) was extracted to compute force descriptors. Corresponding sample entropy (SE) and DFA alpha exponent (DFAα) values are shown for each knee. See the Methods section for details

*Quadriceps force assessment*: As previously described [20], a maximal voluntary isometric test was conducted using an isokinetic dynamometer (HUMAC NORM II^®^). Torque signals were recorded at 1 kHz via an analog/digital converter (EMG830c^®^). Participants were seated with the hip at 85 degrees and the knee at 60 degrees angle, stabilized with inelastic bands. After warm-up, they performed three strong knee extensions (3–5 s each), with the trial showing the peak torque selected for analysis (Fig. 1, upper panel).

Torque data were filtered using a fourth-order low-pass Butterworth filter with a 50 Hz cutoff frequency. Force steadiness and complexity are typically computed from a relatively steady segment of the torque signal, avoiding the initial and final phases of contraction. The size of the segment varied according to the test procedure, ranging from 1.5 to 5 s [20, 21]. In this study, the region of interest (ROI) was selected from the central, 2-s plateau of the torque signal (Fig. 1, lower panels, see gray shading) and was defined as such that it did not necessarily include the instantaneous peak value. This ROI was used for subsequent analyses.

Force magnitude was quantified as average torque (AT), calculated as the mean value within the region of interest. Force control was described by steadiness and complexity. Force steadiness was expressed as the coefficient of variation (CV; the standard deviation relative to the mean, in percentage). Force complexity was assessed via two metrics: temporal regularity, expressed as sample entropy (SE), computed with an embedded dimension (*m*) of 2 and a tolerance (*r*) of 0.1, as in previous investigations [20, 21]; and torque fractal scaling, or long-term statistical dependency, estimated using the detrended fluctuation analysis alpha exponent (DFA alpha [21]). For DFA, window sizes ranged from 4 to 200, divided into 22 non-overlapping bins. All data processing was performed in Python 3.13.5. Sample entropy and the alpha exponent were computed using the *nolds* 0.6.2 package [35].

### Statistical analysis

Missing force signals data and outliers (4% of total dataset) were handled using imputation via multiple imputation by chained equation (MICE) algorithm [36], implemented in Python with the *statsmodels* 0.14.0 package. For two participants, 30-s sit-to-stand test data could not be collected owing to technical issues; these missing values were imputed using the group mean.

Although half of data vectors (54% of variables) deviated from a Gaussian distribution (Shapiro–Wilk’s *P* < 0.014), a three-way mixed model analysis of variance (ANOVA) was used to assess main effects and interactions for sex (male/female), side (involved versus contralateral knee), and previous surgery status (presence/absence of prior surgery in the contralateral knee). ANOVA was selected owing to its reported robustness to violations of normality and its ability to handle unequal group sizes, as supported by prior research [37, 38]. This approach was further supported by a post hoc power analysis (G*Power 3.1.9.7) using the effect sizes obtained in the present study (*f* ranging from 0.32 to 0.72). For sample sizes of 48 and alpha of 0.05, the analysis estimated a statistical power consistently exceeding 90% across this range of effect sizes.

The association between force descriptors—separately for each knee—was assessed using Spearman’s correlation analysis. Correlation coefficients (rho) were interpreted as small (< 0.20), moderate (0.20–0.50), or large (> 0.50).

Finally, the association between force descriptors and functional performance was assessed using linear regression models. Each functional test was entered as the dependent variable in separate models. Force descriptors, expressed as lateral symmetry index (LSI; calculated as involved knee values/contralateral knee values multiplied by 100), were applied as independent variables. Beta standardized coefficients and 95% confidence intervals were computed to quantify the magnitude and direction of associations.

The statistical significance threshold was set at 5%. The false discovery rate (FDR) approach [39] was used to correct for multiple comparisons in the Spearman correlation analysis of force descriptors. All analyses were performed in JASP 0.19.3 and Python environment (using *pingouin* 0.5.5 and *statsmodels* 0.14.4 packages).

## Results

### Participants

A total of 48 patients (31 females and 17 males), scheduled for unilateral TKA (54% in the right knee), were assessed. Their mean (range) age was 64 (41–78) years, height was 161 (145–181) cm, weight was 85 (52–116) kg, and body mass index (BMI) was 32.8 (20.3–46.2) kg/m^2^. Collectively, 12 patients (26%) reported falls in the preceding 6 months, and 39 patients (85%) were currently taking medication, with 30 using polypharmacy (more than two medications).

### Association among force descriptors

**Fig. 2:**
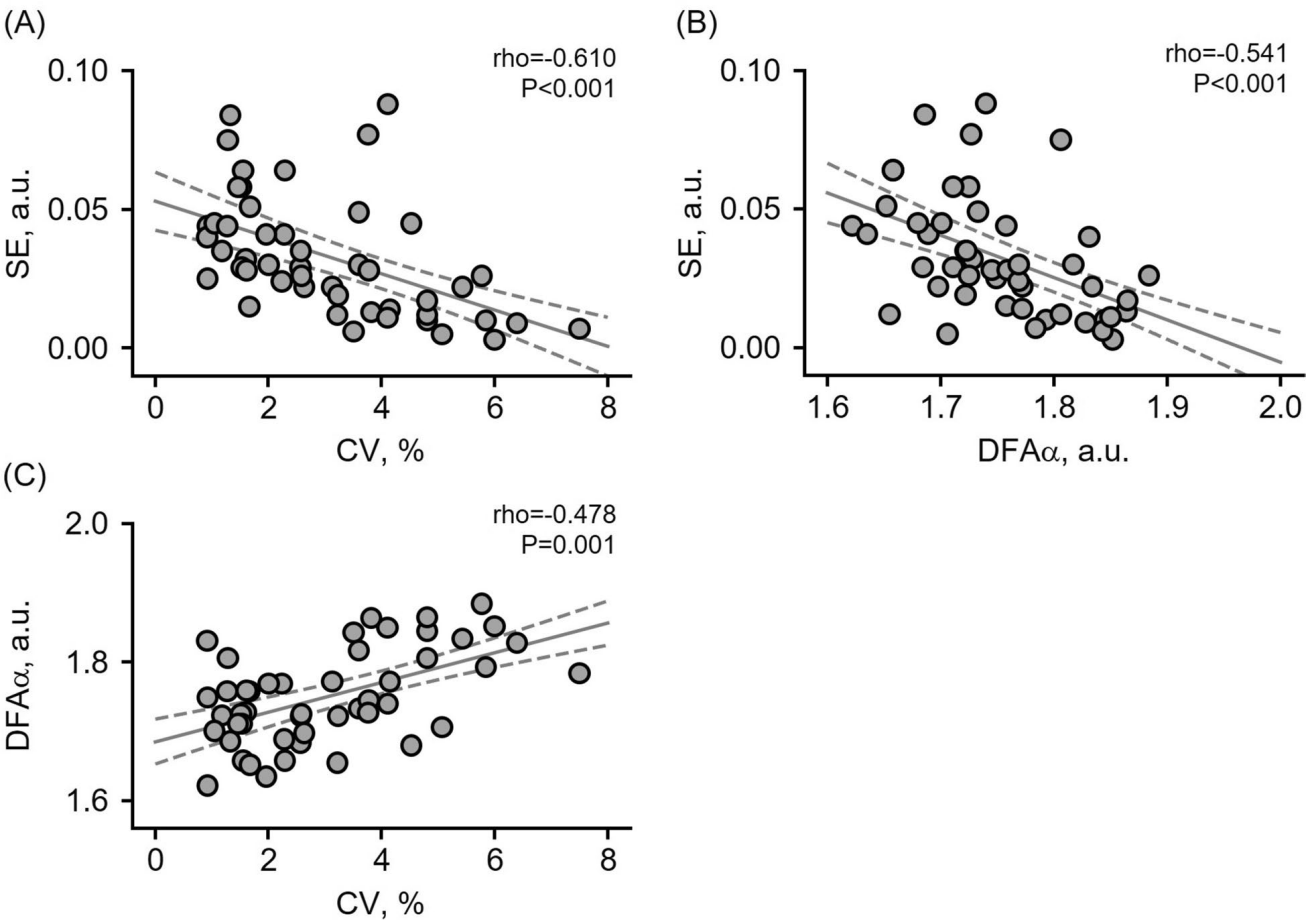
Relationships between force control variables for the involved knee. Panels (**A**) and (**B**) show the negative associations between sample entropy (SE) and the coefficient of variation (CV) and between SE and the DFA alpha exponent (DFAα), respectively. Panel (**C**) displays the positive association between DFAα and CV. For visualization, a linear regression line (solid gray) and its 95% confidence interval (dashed gray) are included. Correlation results (rho and *P*-values) are reported in the inset

The correlation coefficient analysis of force descriptors revealed a consistent pattern for both the involved and contralateral knees. Overall, average torque showed no association with force control variables (all *P* > 0.110). In contrast, sample entropy exhibited large negative association with both the coefficient of variation and the DFA alpha exponent (*P* < 0.001), while the DFA alpha exponent demonstrated a moderate positive association with the coefficient of variation (*P* ≤ 0.001). Figure 2 shows the key relationship for the involved knee.

### Comparison between involved and contralateral knee

The statistical analysis compared force descriptors primarily between sides (the involved knee scheduled for TKA versus the contralateral knee), with sex and previous surgical history included as additional factors.

As no significant main effect of previous surgery status (*P* > 0.720) or interactions between this and the other factors (*P* > 0.209) were observed, further results are presented on the basis of pooled contralateral knee data (irrespective of surgical history). Consequently, the descriptive statistics in Table 1 are stratified only by sex and knee side.

**Table 1:** Summary of the comparative analysis of force descriptors of the involved knee (schedule for TKA) and the contralateral knee (pooled, irrespective of prior surgical)

	Involved knee	Contralateral knee	Sex (*P*-value, *η*^2^)	Side (*P*-value, *η*^2^)
AT (N·m)			< 0.001, 0.196	< 0.001, 0.074
Female	59.8 (23.3)	76.2 (28.6)		
Male	87.1 (30.1)	109.1 (36.9)		
CV (%)			0.411, 0.008	0.768, < 0.001
Female	3.1 (1.8)	2.7 (1.5)		
Male	2.9 (1.4)	3.3 (2.0)		
SE (a.u.)			0.902, < 0.001	0.041, 0.046
Female	0.032 (0.024)	0.025 (0.013)		
Male	0.035 (0.019)	0.023 (0.016)		
DFAα (a.u.)			0.021, 0.070	0.669, 0.002
Female	1.74 (0.06)	1.72 (0.06)		
Male	1.76 (0.07)	1.77 (0.05)		

Data expressed as mean (SD). *AT* average torque; *CV* coefficient of variation; *SE* sample entropy, *DFAα* DFA alpha exponent, *SD* standard deviation. *P*-values and effect sizes are shown only for the significant main effects (sex and side)

A significant main effect of sex was observed for average torque (*P* < 0.001; *η*^2^ = 0.196) and DFA alpha exponent (*P* = 0.021, *η*^2^ = 0.070), with females exhibiting lower strength and reduced alpha exponent than males. For example, in the involved knee, median (range) average torque values were 60 (11–118) N·m in females versus 87 (38–131) N·m in males, while the DFA alpha exponent was 1.74 (1.62–1.86) a.u. in females versus 1.76 (1.65–1.88) a.u. in males. No significant interaction was found between sex, side, or previous surgery (all *P* > 0.209).

**Fig. 3:**
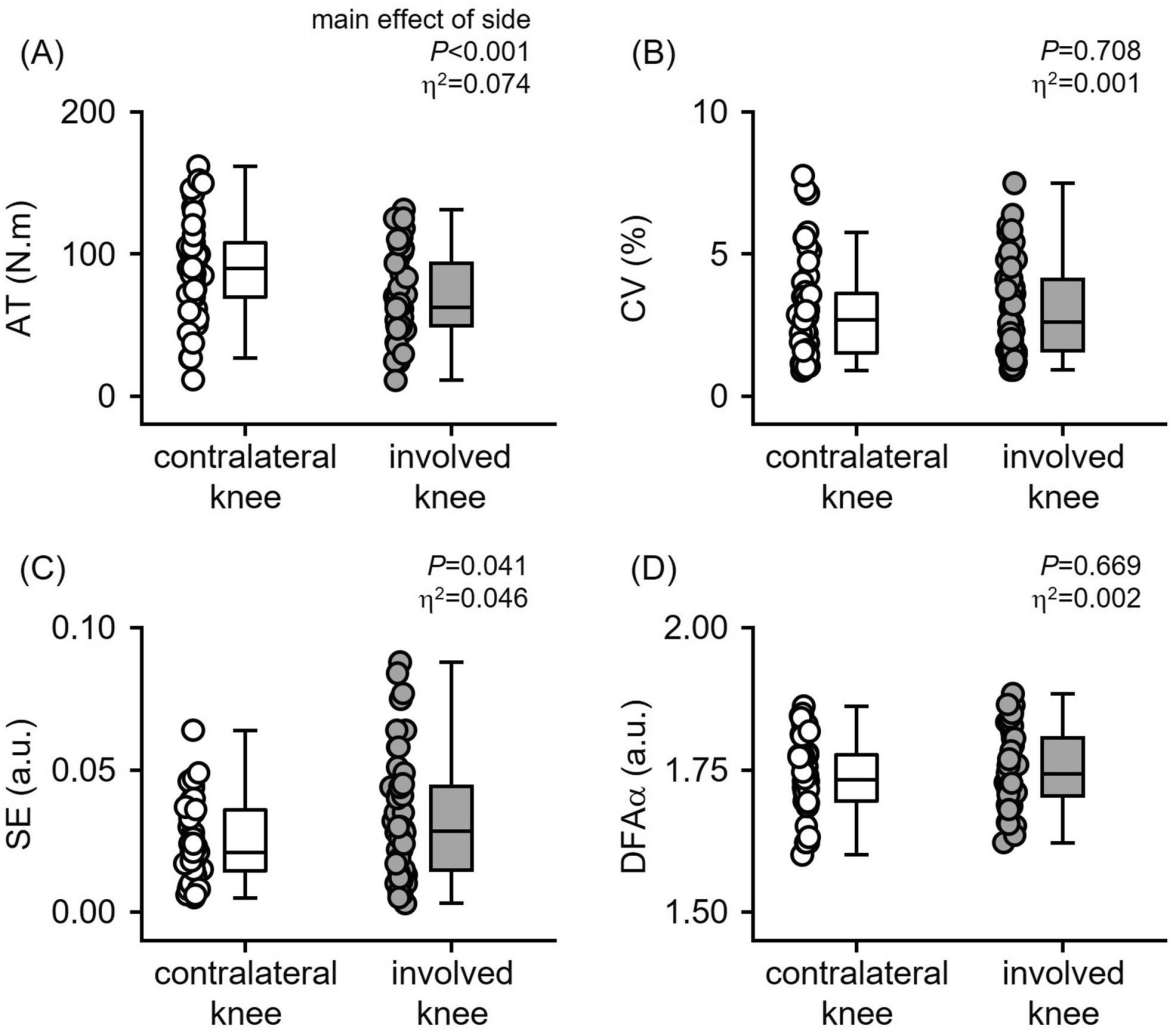
Box plots with overlaid individual data points displaying (**A**) average torque, (**B**) coefficient of variation, (**C**) sample entropy, and (**D**) DFA alpha exponent. Inset values indicate the *P*-values and effect sizes for the main effect of side

A significant main effect of side (involved versus contralateral knee) for average torque (*P* < 0.001, *η*^2^ = 0.074) and sample entropy (*P* = 0.041, *η*^2^ = 0.046) were found. No significant effects of side were found for the coefficient of variation (*P* = 0.768, *η*^2^ < 0.001) or DFA alpha exponent (*P* = 0.669, *η*^2^ = 0.002). The involved knee exhibited lower average torque (Fig. 3A) but greater sample entropy (Fig. 3C) compared with the contralateral knee.

### Association between force descriptors and functional performance

Only average torque and sample entropy were included in the regression model, accounting for inter-knee differences (Fig. 3) and moderate-to-large associations across force control variables (Fig. 2). The linear regression analysis (Table 2) revealed a significant association between average torque LSI and both the sitting-to-standing (*β* = −0.337, *P* = 0.026) and the 30-s sit-to-stand test (*β* = 0.336, *P* = 0.027). No other functional performance measures were significantly associated with either average torque or sample entropy LSI (all *P* > 0.058). Higher muscle strength asymmetry toward the contralateral knee was associated with poorer performance in the sitting-to-standing test and fewer repetitions in the 30-s sit-to stand test.

**Table 2:** Linear regression analysis of force descriptor’s LSI and functional performance

Functional test	LSI, average torque			LSI, sample entropy		
	β^*^	95% CI	*P*-value	β^*^	95% CI	*P*-value
Sitting-to-standing (score)	−0.337	− 0.632, −0.042	0.026	−0.164	−0.459, 0.131	0.269
Single-leg stance (score)	−0.103	−0.411, 0.205	0.505	−0.172	−0.480, 0.136	0.266
Timed Up and Go (s)	−0.290	−0.590, 0.010	0.058	−0.105	−0.405, 0.195	0.485
30-s sit-to-stand (rep./s)	0.336	0.040, 0.631	0.027	0.167	−0.128, 0.463	0.260

^*^Standardized coefficients. *LSI* lateral symmetry index, *rep./s* repetitions per second

## Discussion

In a sample of individuals awaiting unilateral TKA, the involved knee (i.e., the one with end-stage osteoarthritis [OA] clinically requiring replacement) displayed significantly lower average torque and higher sample entropy compared with the contralateral limb, whereas coefficient of variation and DFA alpha showed no side differences. Furthermore, regression analyses revealed that the lateral symmetry index of average torque was positively associated with performance on the 30‑s sit‑to‑stand test and negatively associated with the sitting‑to‑standing score, while sample entropy did not predict any functional outcome. These results indicate that KOA is accompanied by asymmetries between the knee scheduled for replacement and the contralateral knee. It is important to note, however, that the contralateral knee had a heterogeneous and not systematically graded OA status that should not, therefore, be interpreted as a healthy reference. The theoretical and clinical implications of these findings are discussed.

### Force magnitude and control as distinct neuromuscular mechanisms

The correlation analysis reveals a critical dissociation between force magnitude and force control complexity, suggesting that these are governed by distinct neuromuscular mechanisms. This dissociation indicates that a patient can possess good strength yet poor motor control, underscoring the necessity of independent clinical assessment of both domains. In contrast, the strong interrelationships among complexity metrics form a coherent narrative: sample entropy’s negative correlation with both coefficient of variation and the DFA alpha exponent suggests that a more complex, adaptive control strategy (i.e., high entropy) results in lower, more desirable variability (lower coefficient of variation) and a less persistent output (i.e., lower DFA alpha [40, 41]). The observed positive correlation between the DFA alpha exponent and coefficient of variation reinforces this interpretation. These findings are consistent with the suggestion that complexity-based measures are superior markers for detecting subtle motor control impairments missed by traditional strength or variability measures alone (reviewed in [25, 26]), particularly in pathological states characterized by a loss of system complexity [42].

### Sex differences in force control measures

The observed significant main effect of sex, with females exhibiting lower average torque and a slightly reduced DFA alpha exponent compared with males, aligns with and expands upon the existing body of literature on sex-specific neuromuscular function in knee extensors. The well-established strength disparity is largely attributed to physiological factors like lower muscle mass and smaller type II fiber cross-sectional areas rather than activation deficits [43]. The reduced DFA alpha exponent is a more novel finding, indicating a less persistent, more complex torque output that reflects a less rigid control strategy [40, 41]. These findings partially aligns with studies showing that complexity-based measures can reveal sex differences in control strategies where traditional metrics do not [44]. The combination of lower strength and increased complexity suggests a potential neuromuscular “vulnerability” in females, which could contribute to a higher risk of knee pathologies in this population.

### End-stage KOA effects in force magnitude and complexity

The observed reduction in quadriceps strength of the involved knee aligns with the well‑established notion that muscle weakness contributes to KOA progression and functional limitation [8, 45]. The increase in sample entropy, however, suggests a shift toward more irregular, less predictable force output in the affected limb, possibly reflecting altered motor unit recruitment or compromised proprioceptive feedback, as found earlier in elderlies and those with ACL injuries [20, 40]. The elevated sample entropy contrasts with some earlier work reporting reduced complexity in older or frail populations [22, 23], suggesting that knee orthopedic conditions may provoke a distinct neuromuscular control, characterized by heightened complexity.

Studies examining force steadiness in KOA have produced mixed results, often limited by methodological heterogeneity. Because of variations in contraction regimens (isokinetic concentric and/or eccentric, and isometric), intensities (absolute or relative submaximal contractions), and steadiness measures (absolute error, standard deviation, or coefficient of variation), studies have reported smaller [17], larger [15], and smaller or unchanged (depending on the steadiness measure [16]) force steadiness in the involved knee of patients with KOA compared with the dominant knee of a healthy control group. To our knowledge, this is the first study to investigate between-knee asymmetries in force steadiness in patients with KOA.

Importantly, the lack of association between force complexity and functional performance implies that, despite detectable changes in neuromuscular dynamics, the tasks examined (e.g., sit‑to‑stand) may rely predominantly on maximal strength rather than fine‑grained force regulation. Previous investigation [18] similarly found no association between knee adduction moment and force steadiness during submaximal contractions in patients with KOA. Consequently, the principal findings—lower average torque and higher sample entropy in the diseased knee, together with the exclusive link between strength symmetry and functional scores—underscore strength asymmetry as the dominant determinant of functional capacity in this population.

Despite the fundamental differences in muscle adaptation mechanisms between chronic degenerative diseases such as KOA and acute injuries such as ACL rupture, the similarities in their neuromuscular responses in maximal voluntary contractions are remarkable—these include elevated sample entropy [20, 24] and an absence of between-knee differences in force steadiness [20]. The observed similarities, despite different underlying causes, point toward a common pathway of neuromuscular dysfunction, possible due to features such as arthrogenic muscle inhibition [6, 46], disrupted sensory feedback [47, 48], and altered motor pathways structure and function (reviewed in [49]) that occurs with both joint degeneration and trauma.

Altogether, this study demonstrates that end-stage KOA, when clinically indicated for TKA, is characterized by significant alterations in neuromuscular output not only by reducing strength but also by impairing movement predictability, implications that may necessitate revised rehabilitation approaches. However, despite the ability of complexity metrics to identify these subtle changes, strength asymmetry persists as the chief driver of clinical function in advanced disease.

### Study limitations

The study limitations should be considered. Most critically, the lack of a healthy control group means our contralateral knees are not confirmed as functionally normal—our findings show impairment in the TKA knee relative to the less affected side but they cannot define the absolute deficit of end-stage OA versus health, potentially underestimating the true loss. Second, the generalizability of the results is limited by its sample of patients scheduled for unilateral TKA. Third, the pre-operative assessment timing may have allowed acute factors like pain or medication to influence the results. Fourth, the characterization of the study knees was limited: (1) for the involved limb, the duration of end-stage OA was not documented, which is a potential confounder as longer disease history may correlate with greater impairment and (2) for the contralateral knee, data on the time since prior surgery and consistent radiographic or symptomatic criteria were lacking, limiting the precision of its classification. Finally, the focus on gross motor tasks and maximal isometric contractions has inherent boundaries, as this approach potentially misses nuanced deficits detectable with clinical assessments and cannot evaluate submaximal force control or dynamic stability, which are critical for daily activities.

Despite these limitations, important methodological strengths help mitigate their impact: (1) the restricted sample provided a clinically relevant and homogeneous group; (2) the focus on maximal contractions enabled standardized assessment and direct comparison with prior literature on quadriceps torque; and (3) the comprehensive evaluation of neuromuscular function, including both traditional and nonlinear measures, offered novel insights beyond most previous studies.

## Conclusions

This study found that the end-stage osteoarthritic knee had significantly weaker and more complex quadriceps force output than the contralateral knee. Only the asymmetry in strength—not complexity—predicted patients’ functional performance. We also identified novel sex differences in neuromuscular control. Importantly, these outcomes were independent of the contralateral knee’s own symptomatic or surgical status, reinforcing that it should not be considered a healthy control.

## Data Availability

The data that support the findings of this study are available upon reasonable request.

## Abbreviations

ACL: Anterior cruciate ligament
AT: Average torque
CV: Coefficient of variation
DFA: Detrended fluctuation analysis
KOA: Knee osteoarthritis
LSI: Lateral symmetry index
SE: Sample entropy
TKA: Primary total knee arthroplasty
